# Association of a Zero-Separation Neonatal Care Model With Stress in Mothers of Preterm Infants

**DOI:** 10.1001/jamanetworkopen.2022.4514

**Published:** 2022-03-28

**Authors:** Nicole R. van Veenendaal, Anne A. M. W. van Kempen, Birit F. P. Broekman, Femke de Groof, Henriette van Laerhoven, Maartje E. N. van den Heuvel, Judith J. M. Rijnhart, Johannes B. van Goudoever, Sophie R. D. van der Schoor

**Affiliations:** 1 Department of Pediatrics and Neonatology, OLVG, Amsterdam, the Netherlands; 2 Amsterdam UMC, University of Amsterdam, Vrije Universiteit, Emma Children’s Hospital, Amsterdam, the Netherlands; 3 Department of Psychiatry, OLVG, Amsterdam, the Netherlands; 4 Department of Psychiatry, Amsterdam UMC, Vrije Universiteit, Amsterdam, the Netherlands; 5 Department of Pediatrics and Neonatology, NoordWest ZiekenhuisGroep, Alkmaar, the Netherlands; 6 Department of Epidemiology and Data Science, Amsterdam Public Health Research Institute, Amsterdam UMC, Vrije Universiteit, Amsterdam, the Netherlands

## Abstract

**Importance:**

Active participation in care by parents and zero separation between parents and their newborns is highly recommended during infant hospitalization in the neonatal intensive care unit (NICU).

**Objective:**

To study the association of a family integrated care (FICare) model with maternal mental health at hospital discharge of their preterm newborn compared with standard neonatal care (SNC).

**Design, Setting, and Participants:**

This prospective, multicenter cohort study included mothers with infants born preterm treated in level-2 neonatal units in the Netherlands (1 unit with single family rooms [the FICare model] and 2 control sites with standard care in open bay units) between May 2017 and January 2020 as part of the AMICA study (fAMily Integrated CAre in the neonatal ward). Participants included mothers of preterm newborns admitted to participating units. Data analysis was performed from January to April 2021.

**Exposures:**

FICare model in single family rooms with complete couplet-care for the mother-newborn dyad during maternity and/or neonatal care.

**Main Outcomes and Measures:**

Maternal mental health, measured using the Parental Stress Scale: NICU (PSS-NICU). Secondary outcomes included survey scores on the Hospital Anxiety and Depression Scale, Postpartum Bonding Questionnaire, Perceived Maternal Parenting Self-efficacy Scale, and satisfaction with care (using EMPATHIC-N). Parent participation (using the CO-PARTNER tool) was assessed as a potential mediator of the association of the FICare model on outcomes with mediation analyses.

**Results:**

A total of 296 mothers were included; 124 of 141 mothers (87.9%) in the FICare model and 115 of 155 (74.2%) mothers in SNC responded to questionnaires (mean [SD] age: FICare, 33.3 [4.0] years; SNC, 33.3 [4.1] years). Mothers in the FICare model had lower total PSS-NICU stress scores at discharge (adjusted mean difference, −12.24; 95% CI, −18.44 to −6.04) than mothers in SNC, and specifically had lower scores for mother-newborn separation (adjusted mean difference, −1.273; 95% CI, −1.835 to −0.712). Mothers in the FICare model were present more (>8 hours per day: 105 of 125 [84.0%] mothers vs 42 of 115 [36.5%]; adjusted odds ratio, 19.35; 95% CI, 8.13 to 46.08) and participated more in neonatal care (mean [SD] score: 46.7 [6.9] vs 40.8 [6.7]; adjusted mean difference, 5.618; 95% CI, 3.705 to 7.532). Active parent participation was a significant mediator of the association between the FICare model and less maternal depression and anxiety (adjusted indirect effect, −0.133; 95% CI, −0.226 to −0.055), higher maternal self-efficacy (adjusted indirect effect, 1.855; 95% CI, 0.693 to 3.348), and better mother-newborn bonding (adjusted indirect effect, −0.169; 95% CI, −0.292 to −0.068).

**Conclusions and Relevance:**

The FICare model in our study was associated with less maternal stress at discharge; mothers were more present and participated more in the care for their newborn than in SNC, which was associated with improved maternal mental health outcomes. Future intervention strategies should aim at reducing mother-newborn separation and intensifying active parent participation in neonatal care.

**Trial Registration:**

Netherlands Trial Register identifier NL6175

## Key Points

**Question:**

Is there an association between the neonatal care setting—a family integrated care (FICare) model in single family rooms with complete couplet-care for the mother-newborn dyad vs standard neonatal care in open bay units—and mental health and participation outcomes among mothers of preterm newborns?

**Findings:**

In this cohort study of 296 mothers of preterm infants, mothers reported experiencing less stress and participated more when they and their infants received care in wards using a FICare model. Participation in infant care mediated the beneficial association of the FICare model and mothers’ depressive symptomatology, self-efficacy, and mother-newborn bonding.

**Meaning:**

These findings suggest that intervention strategies aimed at reducing mother-newborn separation and intensifying active maternal participation are warranted.

## Teaser

This cohort study of Dutch mothers with infants born preterm compares stress and participation levels of mothers treated in family integrated care units with mothers receiving standard neonatal care.

## Introduction

Having a preterm infant (born before 37 weeks of gestation) in the neonatal intensive care unit (NICU) can be a stressful experience, and parents of preterm infants are at a higher risk of developing depression and anxiety postnatally.^1,2,3^ The experience of parents who have an infant hospitalized in the NICU can be traumatic, and may result in some developing posttraumatic stress complaints.^4,5^ They are generally assigned to a supportive role during their infant’s hospital stay and often feel insecure or unprepared to care for their infant after discharge.^6,7,8,9^ Additionally, because of hospital policies and accommodations, parents often cannot be with their infant continuously, leading to parent-infant separation during maternal and neonatal care.^10,11,12,13^

Changing hospital care culture to enable parents to actively participate in care, be present continuously, and achieve closeness with their newborns can be challenging.^8,14,15,16^ Previous studies have shown that participation in care with a family integrated care (FICare) approach can alleviate maternal stress at discharge.^17,18^ Also, in 2 systematic reviews and meta-analyses, NICUs with single family rooms (SFRs) were associated with health benefits for infants^19^ and parents, specifically stress reduction in mothers,^10^ which is possibly due to an increased parental presence, skin-to-skin care, and involvement in care.^20^ However, the exact mechanisms on how FICare and SFRs accommodate a reduction in stress and what exact domains of participation in care are promoted and need further reinforcement remains to be elucidated.^10,21^ Also, as not all units are able to change their architectural setting to a SFR design and because FICare can be implemented in open bay units, it is important to discern if active parent participation is a mediator for maternal mental health outcomes (such as anxiety and depression).

This cohort study was intended to explore the association of a FICare model in SFRs with stress in mothers of preterm infants compared with standard neonatal care (SNC) in open bay units. Secondary objectives were to determine if the FICare model was associated with improved outcomes in maternal depression, self-efficacy, mother-newborn bonding, and satisfaction with care. We studied active participation in neonatal care as a potential mediator in the pathway between the FICare model and maternal mental health outcomes.

## Methods

This study is part of the AMICA study (fAMily Integrated CAre in the neonatal ward study), a prospective observational cohort study comparing an innovative neonatal care model (FICare model) with standard neonatal care in open bay units (eMethods in the Supplement). The primary outcome of this study was to track neurodevelopment in preterm infants at 2 years of corrected age. The mental health of parents was also studied in both the short- and longer-term. The study was registered on December 23, 2016, in the Netherlands Trial Registry (NL6175). Hospital architectural design limited randomization between hospitals, and randomization within hospitals was impossible given the risk of cross-contamination. Therefore, we included infants consecutively who were admitted to participating units. This study followed the Transparent Reporting of Evaluations With Nonrandomized Designs (TREND) reporting guideline and A Guideline for Reporting Analyses of Randomized Trials and Observational Studies-Short Form (AGReMA-SF) checklist for reporting mediation analyses.^22,23^ This study was approved by the medical ethical review committee of Medical Research Ethics Committees United Nieuwegein, the Netherlands; participating parents provided written informed consent.

All infants born in or transferred to the level-2 neonatal units participating in the study (1 exposure and 2 control sites) in the Netherlands were eligible. All participating units had a comparable patient population. Preterm infants (defined as infants born before 37 weeks’ gestation) with a hospital stay longer than 7 days and their parents were included after the parents provided informed consent. For this study, we analyzed the mothers of the families. Exclusion criteria were severe psychosocial problems (parents with active psychiatric illness [ie, psychosis] and/or under supervision of child services), parents not proficient in Dutch or English, infant congenital abnormalities likely to influence neurodevelopment, and if death of an infant occurred (see eMethods in the Supplement).

### Exposure (FICare Model)

The exposure setting comprised several aspects, including implementation of FICare principles^18^ with active parent participation and collaboration between the parents and health care team and the integration of neonatal and maternity wards to enable couplet-care in SFRs.^10,24^ The mother-child center was opened in October 2014 in a large teaching hospital in Amsterdam, the Netherlands, with 53 SFRs and full integration of maternity and neonatology services.^24^ Mothers and infants always stayed together in 1 SFR and never had to be separated, as couplet-care can be provided when both needed medical care. Fathers or partners were able to sleep in the SFR and were welcome 24 hours a day.^24^ In these rooms, prenatal monitoring, labor, and postnatal care could be provided for mother and infant together (eFigure 1 in the Supplement). Additionally, a concomitant FICare program was implemented in which parents were trained to be their infant’s primary caregiver while nurses supported, taught, coached, and counseled parents and performed specific nursing tasks^9,18,25^ and necessary specialized medical care, such as cardiorespiratory monitoring, intravenous fluids or antibiotics, placing nasogastric tubes, noninvasive and short-term ventilation, and phototherapy. Parents were encouraged but not obliged to actively participate in their infant’s care and be present 6 to 8 hours per day.^18^ Parents could actively participate as much as they felt comfortable with in neonatal care by (for instance, and not limited to) providing feedings by nasogastric tube, bottle or breast, providing skin-to-skin care, weighing, and temperature regulation. Family-centered rounds were implemented that included parents on medical rounds, involving them in patient management, and enabling them to hear first-hand the developments in their infant’s condition. Parents could provide information on their infant’s general well-being, ask questions, and participate in shared decision making.^26,27^

### Control Group (SNC)

SNC in open bay units (OBUs) was provided in 2 different level-2 neonatal units in Alkmaar and Amsterdam, The Netherlands. These units had an open configuration with newborns staying together in 1 unit (with a maximum of approximately 18 infants admitted simultaneously) (eFigure 2 in the Supplement). These OBUs were close to the maternity ward, but physically separated. Infants who required high-intensive care, tubefeeding, cardiorespiratory monitoring, respiratory support, antibiotics, or phototherapy were admitted to these wards. Adjacent to these wards were maternity wards where mothers could stay up to 7 days after giving birth. Parents could be with their infant, provide skin-to-skin care and (breast-)feeding, and participate in their infant’s care. Medical rounds were done in a separate room without parents. Nurses provided routine care. The OBUs could not provide the necessary facilities for parents to be present 24 hours, especially because they lacked a place to sleep or rest for the mother. Facilities in the OBU included: a comfortable chair at bedside, equipment to express breastmilk near the infant, and separate rooms to have conversations with the medical team.

### Outcomes and Mediators

The predefined primary outcome for this study was maternal stress as measured by the Parental Stress Scale: NICU (PSS-NICU) questionnaire^28^ at discharge. Parents rated their experiences of stressors associated with the hospitalization of their child on a 5-point rating scale ranging from “not at all stressful” (scored as 0) to “extremely stressful” (5),^28^ for a maximum score of 130, with higher scores indicating more stress. Secondary maternal mental health outcomes included: measurements at discharge of maternal depressive symptoms and anxiety using the Hospital Anxiety and Depression Scale (42-point maximum, with higher scores indicating more depressive symptoms),^29^ parent self-efficacy with the Perceived Maternal Parenting Self-efficacy Scale (80-point maximum, with higher scores indicating more self-efficacy),^30^ impaired mother-newborn bonding using the Postpartum Bonding Questionnaire (125-point maximum, with higher scores indicating more impaired mother-newborn bonding),^31^ satisfaction with care and empowerment using EMPATHIC-N (EMpowerment of PArents in THe Intensive Care–Neonatology) (6-point scale, with higher scores indicating more satisfaction).^32^ Mothers filled out how they participated and collaborated with health care staff in neonatal care using the CO-PARTNER tool (62-point maximum, with higher scores indicating more participation and collaboration in neonatal care^21^).

Also, mothers filled out a general questionnaire with details on their education, current job, and the cultural background they identified most with (classified by the participant). To improve response rates, mothers were reminded up to 2 times (7 and 14 days after initial questionnaires were sent) (see eMethods and eTable 1 in the Supplement).

### Statistical Analysis

Two-sample *t* tests were used to compare continuous variables between the FICare group and SNC group. Mann-Whitney *U* tests were used for nonnormally distributed variables. To analyze proportions between groups the χ^2^ test was used. If expected cell counts were 5 or less, we calculated differences with the Fisher exact test.

Baseline characteristics between mothers with and without outcome variables at discharge were compared. We assumed that the data were missing-at-random. The proposed guidance as explained by Sterne et al^33^ was applied for missing data, and we applied the multivariate imputation by chained equations (mice) procedure with parcel mean summary scores to missing data at the item level.^34^ All variables used in the analyses were included in the imputation model, as well as auxiliary variables related to the probability of missing data or to the variables with missing data itself. Variables that were multicollinear with other included variables were excluded from the imputation model. For all data sets, we performed 10 imputations and 50 iterations to obtain imputed data sets. Convergence was checked graphically with convergence plots. All analyses were performed on the imputed data sets and results were pooled by using Rubin Rules.^35^

We performed multivariable linear and logistic regression in imputed data sets estimating crude and adjusted associations between the FICare model and maternal mental health outcomes. Logarithmic transformations were applied to normalize skewed distributions, or, if unsuccessful, dichotomization. Potential confounders and effect modifiers were identified from the literature and assessed using statistical analyses (eMethods in the Supplement).

**Figure 1.**
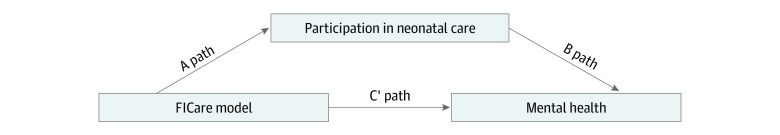
Parent Participation as a Mediator of the Association of the Family Integrated Care (FICare) Model With Maternal Mental Health

We hypothesized that the FICare model (exposure) transmits its association on maternal mental health outcomes (the outcome) at discharge (as a partial effect) through active parent participation (the mediator, CO-PARTNER score) (Figure 1). Mediation analyses on the imputed data set were therefore applied to analyze, identify, and explain the underlying mechanisms of the observed association of the FICare model on mental health outcomes in mothers (ie, the *c*-path)^36^ also in the absence of a significant total association (*c*-path) as described before.^37^

In addition to the total association model, 2 linear regression models were fitted. Total parent participation was included in single mediator models as an individual potential mediator of different mental health outcomes in mothers (Figure 1). In the first regression model, the association of the FICare model on the mediator was estimated (*a*-path). In the second regression model, the association of the mediator (ie, participation) on outcomes (*b-*path) and the direct effect of the FICare model on outcomes (*c’*-path) were estimated. We calculated the indirect effect (the amount of mediation) in the single mediator models as the product of the *a* and *b* coefficients. Crude and adjusted mediation analyses were performed. In the adjusted analyses, confounders were added to all models. We used bootstrap 95% CIs based on 1000 bootstrap resamples around the indirect effects.^38,39^

We used R version 3.6.1 for statistical analyses (R Project for Statistical Computing),^40^ including the mice package for multiple imputation,^41^ the VIM package for analyzing missing data patterns,^42^ and the boot package for the bootstrap 95% CIs.^43^ For all tests, *P* < .05 was considered statistically significant. Data analysis was performed from January to April 2021.

## Results

**Figure 2.**
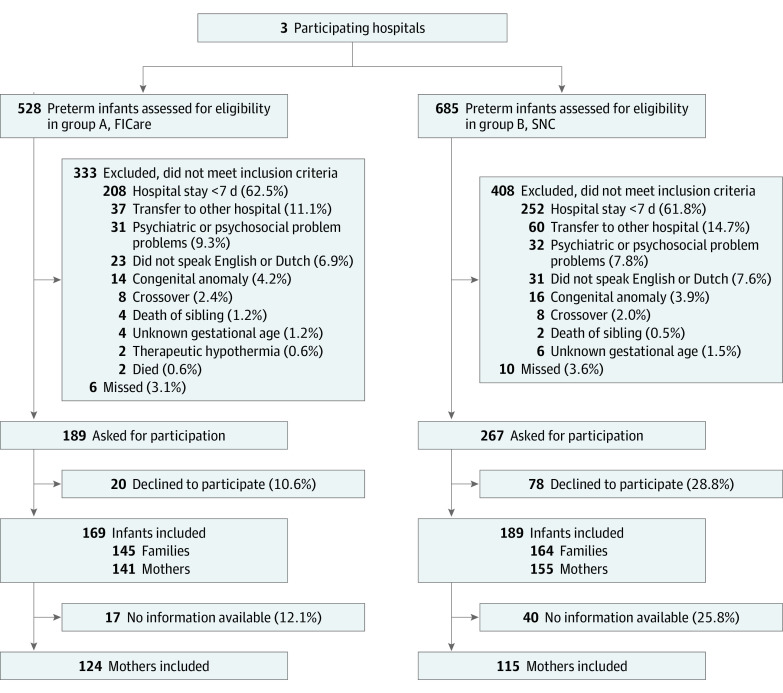
Flow Diagram of Study FICare indicates family integrated care; SNC, standard neonatal care. Missing data and follow-up can be found in the eTable 5 in the Supplement.

From May 19, 2017, through January 8, 2020, we recruited 309 families (145 in FICare and 164 in SNC), encompassing 358 infants and their parents (Figure 2). During the recruitment period, one of the control sites changed to a double-bed occupancy with SFR-like design and FICare practices; this site discontinued recruitment of control patients in March 2019. Two hundred ninety-six mothers (95.8%) consented to participate in the study regarding their mental health (141 in FICare and 155 in SNC), and 239 mothers (80.7%) filled out surveys and were analyzed. A total of 124 mothers in the FICare model were analyzed (mean [SD] age, 33.3 [4.0] years) and 115 mothers in SNC control group were included in analysis (mean [SD] age, 33.3 [4.1] years) (response rates and missing data available in eTables 2-5 in the Supplement).

Baseline characteristics for mothers were similar between the exposure and control groups with the exception of infant gestational age, which was lower in the FICare model (median [IQR] age, 32 weeks, 1 day [29 weeks 3 days to 34 weeks 5 days] vs 34 weeks [32 weeks 2 days to 34 weeks 6 days]; *P* < .001, Mann-Whitney *U* Test) (Table 1). Infants were also less often born in the level-2 facility in the FICare model compared with SNC (53 of 124 [42.7%] vs 80 of 115 [69.6%]; *P* < .001, χ^2^ test).

**Table 1.** Baseline Characteristics of Mothers Participating in Study

Characteristic	Participants, No./total No. (%)^a^		*P* value
	FICare group (n = 124)	SNC group (n = 115)	
Age, mean (SD), y	33.3 (4.0)	33.3 (4.1)	.56
University degree	108/113 (95.6)	89/100 (89)	.19
Paid job	91/113 (80.5)	85/100 (85)	.73
Identifies with Dutch cultural background	87/115 (76)	89/102 (87)	.05
Stress of pregnancy, mean (SD)^b^	2.3 (1.3)	2.3 (1.2)	.95
Stress of birth, mean (SD)^b^	3.0 (1.4)	2.8 (1.3)	.24
Preeclampsia	29/120 (24)	21/114 (18)	.36
HELLP syndrome	5/124 (4)	7/112 (6)	.70
Use of psychotropic drugs	2/124 (2)	3/115 (3)	>.99^c^
Gestational age, median (IQR) [range]	32 wk 1 d (29 wk 3 d to 34 wk 5 d) [24 wk 1 d to 36 wk 6 d]	34 wk (32 wk 2 d to 34 wk 6 d) [24 wk 1 d to 36 wk 6 d]	<.001
Born <32 wk of gestation	60/124 (48)	25/115 (22)	<.001
Inborn infant (born in level-2 hospital)	53/124 (43)	80/115 (70)	<.001
Singleton pregnancy	103/124 (83)	102/115 (89)	.29
Vaginal delivery	64/124 (52)	60/115 (52)	.30
First child upbringing	81/113 (72)	65/95 (68)	.13
Plan for upbringing together with partner	107/113 (95)	86/94 (91)	.11
Total stress at admission, mean (SD)^d^	55.7 (22.7)	56.1 (21.8)	.88
Depression and anxiety score at admission, median (IQR)^e^	10 (8 to 14)	12 (7 to 24)	.46

Abbreviations: FICare, family integrated care; HELLP, hemolysis, elevated liver enzymes, and low platelets count (complication of pregnancy); SNC, standard neonatal care.

^a^ Denominators differ because of missing data (see eTable 5 in the Supplement).

^b^ 5-point scoring scale, with 5 indicating “extremely stressful.”

^c^ Fisher exact test.

^d^ 130-point maximum score, with higher score indicating more stress.

^e^ 42-point maximum score, with higher score indicating more depressive symptoms.

Overall, mothers in the FICare model had significantly lower total NICU stress scores (adjusted mean difference, −12.24; 95% CI, −18.44 to −6.044), lower stress from infant behavior, sights, and sounds (adjusted mean difference, −5.819; 95% CI, −10.29 to −1.350), and lower stress scores due to parental role alteration (adjusted mean difference, –6.423; 95% CI, −8.910 to −3.937) at discharge compared with mothers in SNC (Table 2). In the PSS-NICU questionnaire, 34 of 188 mothers (18.1%) scored their stress due to separation from their infant as extremely stressful (eTable 6 in the Supplement), and the majority of these responses were given by mothers in SNC (24 of 34 [70.6%]). The mean (SD) stress score on this item was significantly lower in the FICare model (2.1 [2.0]) compared with mothers with infants admitted to SNC (3.3 [1.6]), a result that held after adjusting for confounders (adjusted mean difference, −1.273; 95% CI, −1.835 to −0.712).

**Table 2.** Maternal Participation in Neonatal Care During Hospital Stay and Mental Health Outcomes at Discharge^a^

Measure	Mean (SD)		Mean difference (95% CI)	*P* value	Adjusted mean difference (95% CI)^b^	*P* value
	FICare (n = 124)	SNC (n = 115)				
**During hospital stay**						
Presence						
Median (IQR), h/d	20 (9 to 24)	6 (4 to 12)	NA^c^	NA	NA^c^	NA
>8 h/d, No. (%)	105 (84.7)	42 (36.5)	9.578 (4.988 to 19.39)^d^	<.001	19.35 (8.130 to 46.08)^d^	<.001
Total participation (maximum score 62)	46.7 (6.9)	40.8 (6.7)	5.917 (4.126 to 7.708)	<.001	5.618 (3.705 to 7.532)	<.001
Domain 1, participation in daily care (maximum score 22)	16.5 (4.0)	15.4 (3.1)	1.043 (0.081 to 2.006)	.03	0.953 (−0.061 to 1.969)	.07
Domain 2, participation in medical care (maximum score 8)	4.7 (1.8)	3.5 (1.5)	1.196 (0.754 to 1.638)	<.001	1.037 (0.582 to 1.492)	<.001
Domain 3, information gathering (maximum score 3)	2.3 (0.8)	2.5 (0.6)	−0.190 (−0.402 to 0.022)	.08	−0.311 (−0.537 to −0.085)	.008
Domain 4, advocacy and leadership (maximum score 3)	2.2 (1.0)	1.5 (1.1)	0.692 (0.417 to 0.965)	<.001	0.636 (0.357 to 0.916)	<.001
Domain 5, time spent with infant (maximum score 12)	8.3 (2.4)	6.1 (2.8)	2.157 (1.412 to 2.902)	<.001	2.297 (1.529 to 3.065)	<.001
Domain 6, comforting the infant (maximum score 14)	12.7 (1.3)	11.7 (1.7)	1.021 (0.514 to 1.528)	<.001	1.010 (0.502 to 1.519)	<.001
**Outcomes at discharge**						
NICU stress (maximum score 130)	47.2 (22.2)	57.0 (22.2)	−9.737 (−16.01 to −3.465)	.003	−12.24 (−18.44 to −6.04)	<.001
Behavior and sights and sounds (maximum score 95)	34.5 (16.2)	38.6 (15.2)	−4.022 (−8.721 to 0.677)	.09	−5.819 (−10.29 to −1.350)	.01
Parental role alteration (maximum score 35)	12.7 (8.1)	18.4 (9.1)	−5.715 (−8.239 to −3.191)	<.001	−6.423 (−8.910 to −3.937)	<.001
Being separated from my baby (maximum score 5)	2.1 (2.0)	3.3 (1.6)	−1.174 (−1.698 to −0.651)	<.001	−1.273 (−1.835 to −0.712)	<.001
Anxiety and depression (maximum score 42), median (IQR)	9.8 (5.3 to 15.3)	10.1 (4.8 to 15.5)	−0.062 (−0.252 to 0.128)^e^	.52	−0.117 (−0.308 to 0.075)^e^	.23
Self-efficacy (maximum score 80)	63.7 (8.9)	62.7 (9.0)	1.002 (−1.357 to 3.361)	.40	0.916 (−1.532 to 3.364)	.46
Impaired mother-newborn bonding (maximum score 125), median (IQR)	10.2 (4.1 to 16.3)	9.3 (4.3 to 14.4)	0.142 (−0.076 to 0.361)^e^	.20	0.097 (−0.130 to 0.324)^e^	.40
Satisfaction with care (maximum score 6), median (IQR)	5.6 (5.3 to 5.9)	5.6 (5.2 to 5.9)	0.018 (−0.104 to 0.140)^e^	.77	0.023 (−0.099 to 0.146)^e^	.71

Abbreviation: NICU, neonatal intensive care unit.

^a^ All outcomes are pooled estimates from multiple imputed data sets, Outcomes are calculated from the imputed data sets.

^b^ Adjusted for gestational age, gemelli status, education, age, Dutch background, singleton status, stress at birth, and first child upbringing.

^c^ Regression estimates could not be calculated due to nonnormality, also after logarithmic transformation.

^d^ Odds ratio.

^e^ After logarithmic transformation.

### Participation During Hospital Stay

Mothers in the FICare model were present more than mothers in SNC (median [IQR] hours per day, 20 [9-24] vs 6 [4-12] hours). One hundred five of 124 mothers (84.7%) were able to be present for at least 8 hours in the FICare model compared with 42 of 115 (36.5%) in SNC (adjusted odds ratio, 19.35; 95% CI, 8.13 to 46.08) (Table 2). Mothers in the FICare model participated more in the care of their infant (adjusted mean difference, 5.618; 95% CI, 3.705 to 7.532) compared with SNC (Table 2). Participation was higher for mothers in the FICare model compared with SNC, specifically within daily care, medical care (including tubefeeding, monitoring of the infant, regulation of visitation to the infant and participating in daily rounds), advocacy and leadership, time spent with the infant, and comforting of the infant (total participation mean [SD] score, 46.7 [6.9] vs 40.8 [6.7]; *P* < .001). In the FICare model, mothers required less information compared with mothers in SNC (mean [SD] score, 2.3 [0.8] vs 2.5 [0.6]; *P* = .008).

### Mediation Analyses of Active Parent Participation on Maternal Mental Health Outcomes

With mediation analyses we estimated the indirect effect (the *ab* path) of the FICare model on maternal mental health outcomes through active parent participation. We also estimated the direct effect of the FICare model on maternal mental health outcomes that was not explained by increased active parent participation (through the *c’* path).

Increased active maternal participation was a significant mediator of the association between the FICare model and less maternal depression and anxiety (adjusted indirect effect, −0.133; 95% CI, −0.226 to −0.055) (*ab* path), better mother-newborn bonding (adjusted indirect effect, −0.169; 95% CI, −0.291 to −0.068) (*ab* path) and higher maternal self-efficacy (adjusted indirect effect, 1.855; 95% CI, 0.693 to 3.348) (*ab* path), at discharge (Table 3). In other words, the higher active maternal participation in the FICare model (mean adjusted *a* path [SE], 5.618 [0.969]) was associated with lower depressive symptomatology (mean adjusted *b* path, −0.024 [0.007]), better mother-newborn bonding scores (mean adjusted *b* path, −0.030 [0.009]), and higher self-efficacy scores (mean adjusted *b* path, 0.330 [0.091]). No beneficial direct effects (*c’* paths) were found of the FICare model on maternal depression and anxiety, mother-newborn bonding, and maternal self-efficacy.

**Table 3.** Mediation Analysis of Mothers’ Participation During Infant Hospital Stay and Mental Health at Discharge

Outcome	Association of the FICare model with mediator (participation), *a* pathway, mean (SE)	Association of mediator (participation) with outcome, *b* pathway, mean (SE)	Indirect effect (*ab* pathway), (95% CI)	Association of FICare with outcome, mean (SE)	
				*c’*-Pathway	*c*-Pathway
**Crude analyses**					
Stress	5.917 (0.908)	−0.393 (0.225)	−2.324 (−5.156 to 0.186	−7.410 (3.485)	−9.737 (3.167)
Self-efficacy	5.917 (0.908)	0.343 (0.092)	2.031 (0.805 to 3.479	−1.030 (1.299)	1.002 (1.196)
Satisfaction with care	5.917 (0.908)	0.004 (0.004)	0.024 (−0.025 to 0.078	−0.006 (0.067)	0.018 (0.062)
Depression and anxiety^a^	5.917 (0.908)	−0.024 (0.008)	−0.143 (−0.243 to −0.057	0.081 (0.105)	−0.062 (0.096)
Impaired mother-newborn bonding^a^	5.917 (0.908)	−0.031 (0.009)	−0.186 (−0.316 to −0.077	0.328 (0.120)	0.142 (0.111)
**Adjusted analyses^b^**					
Stress	5.618 (0.969)	−0.382 (0.214)	−2.148 (−5.045 to 0.201	−10.09 (3.397)	−12.24 (3.13)
Self-efficacy	5.618 (0.969)	0.330 (0.091)	1.855 (0.693 to 3.348	−0.939 (1.322)	0.916 (1.242)
Satisfaction with care	5.618 (0.969)	0.007 (0.004)	0.036 (−0.012 to 0.095	−0.013 (0.067)	0.023 (0.062)
Depression and anxiety^a^	5.618 (0.969)	−0.024 (0.007)	−0.133 (−0.226 to −0.055	0.017 (0.101)	−0.117 (0.097)
Impaired mother-newborn bonding^a^	5.618 (0.969)	−0.030 (0.009)	−0.169 (−0.292 to −0.068	0.267 (0.121)	0.097 (0.114)

^a^ Outcomes are pooled estimates from multiple imputed data sets.

^b^ After logarithmic transformation.

The FICare model was associated with less stress for mothers at discharge compared with mothers in SNC. Increased active parent participation in the FICare model was a potential mediator of this association but did not reach statistical significance (adjusted indirect effect, −2.148; 95% CI, −5.045 to 0.201) (*ab* path) (Table 3). The direct effect (*c’* path) of the FICare model on maternal NICU stress remained large after adjustment for active parent participation (mean adjusted *c’* path [SE], −10.09 [3.397]). Parent satisfaction was not different between the FICare model and SNC, and increased active parent participation was not a mediator of the association between the FICare model and satisfaction with care (adjusted indirect effect, 0.036; 95% CI, −0.012 to 0.095).

## Discussion

This study showed that mothers of preterm infants experienced less stress at discharge when admitted to a setting with FICare in SFRs compared with SNC. Mothers in the FICare model were able to be present more and participate more in neonatal care, which was associated with improved mental health outcomes including less depression, better mother-newborn bonding, and higher self-efficacy.

In concordance with previous research, our results indicated an association between mother-newborn separation and high stress levels in mothers of preterm infants^8,13^ admitted to SNC settings. Mother-newborn separation is one of the main challenges health care professionals currently encounter when caring for mothers and infants postnatally, especially when both need medical care. Additionally, during the COVID-pandemic it has become apparent that restrictive policies and mother-newborn separation are of great concern.^44^ Parents have reported that restrictions limit their ability to bond with their infant, to participate in care, and negatively impact breastfeeding as well.^45,46,47^

For NICU stress, a direct association (*c’* path) with the FICare model—independent of active maternal participation—was present. This could indicate that the architectural design with complete couplet-care for the mother-newborn dyad in SFRs was an important factor associated with less maternal stress at discharge, as has been shown before.^10^ The architectural design may have been less important for the other maternal mental health outcomes, since we found no direct beneficial association (*c’* path) for these outcomes. However, increased active maternal participation was a significant mediator of the association between the FICare model and less maternal depression and anxiety, better mother-newborn bonding, and higher maternal self-efficacy. These findings suggest that for maternal depression, mother-newborn bonding, and maternal self-efficacy, specific attention should be pointed toward active maternal partnership and collaboration in neonatal care. Improving active maternal participation and collaboration in neonatal care is feasible independent of the architectural design, as the FICare methodology was initially developed in an OBU.^48,49,50^

Future research should focus on both parental and neonatal outcomes after discharge, as effects of NICU hospitalization on infants (ie, neurodevelopment^19^) and parents (ie, traumatic stress^51^) could persist. Future studies should also explore how hospitalization of a preterm infant affects fathers or partners, as they too can experience adverse outcomes.^52,53,54^ Additional research can also focus on an exact definition of zero separation in this context, as one can still feel emotionally connected without being physically present. For instance, research studies could qualitatively focus on the perception of emotional closeness and the pathways toward emotional closeness that might be facilitated in our FICare model from parents’ perspectives.^55^

### Strengths and Limitations

Strengths of this study included the use of a validated questionnaire (CO-PARTNER)^21^ to evaluate maternal participation in neonatal care, which to our knowledge has not been done as rigorously before.^20,21^ We used advanced statistical techniques for missing data and mediation analyses. We included families with infants within a range of all gestational ages, reflecting the reality of a level-2 neonatal unit, and high response rates were achieved.

As this was a nonrandomized study, there were several limitations that should be considered. We had different enrollment numbers between the FICare model and SNC settings. This was mainly due to nonconsent in SNC and not from missed participants (these numbers were similar between settings). Also, potential baseline differences were present, specifically for gestational age. However, despite this, mothers in the FICare model still reported less stress due to parental role alteration, and specifically less stress from being separated from their infant.

Additionally, the potential causality that might be suggested with mediation analysis should also be considered. Mothers who are less depressed, better bonded, and/or highly self-efficient might also participate more in care, and health care professionals should consider this when implementing programs aimed at increasing parent participation.

## Conclusions

In this study, setting up level-2 neonatal units with a FICare model in single family rooms with complete couplet-care for the mother-newborn dyad was associated with reduced maternal stress at discharge compared with SNC in OBUs with separate maternity care. In the FICare model, mothers could participate and collaborate more in neonatal care, which is associated with ameliorated maternal mental health. For future ward reconfigurations, zero separation between mothers and their newborn should be strived for. However, independent of the architectural design of the neonatal unit, mothers should be allocated as active partners in neonatal care.

## Notes

Supplement.
eMethods.
**eFigure 1.** Images of Care Single Family Rooms **eFigure 2.** Image of Open Bay Unit With Standard Neonatal Care **eTable 1.** Scale Properties and CO-PARTNER Tool **eTable 2.** Response Rates of Mothers **eTable 3.** Baseline Characteristics of Mothers With or Without Filled Out Questionnaires **eTable 4.** Baseline Characteristics of Mothers With or Without Filled Out Questionnaires at Discharge **eTable 5.** Missing Data in Baseline Characteristics **eTable 6.** Answers on the PSS-NICU **eTable 7.** Associations Between Parent Participation and Outcomes
eReferences.
