# Supplementary material for: Association of a Zero-Separation Neonatal Care Model With Stress in Mothers of Preterm Infants
Source: JAMA Netw Open. 2022 Mar 28;5(3):e224514. doi: 10.1001/jamanetworkopen.2022.4514 (PMC8961319; doi:10.1001/jamanetworkopen.2022.4514)
Supplement: Supplement. — eMethods. eFigure 1. Images of Care Single Family Rooms eFigure 2. Image of Open Bay Unit With Standard Neonatal Care eTable 1. Scale Properties and CO-PARTNER Tool eTable 2. Response Rates of Mothers eTable 3. Baseline Characteristics of Mothers With or Without Filled Out Questionnaires eTable 4. Baseline Characteristics of Mothers With or Without Filled Out Questionnaires at Discharge eTable 5. Missing Data in Baseline Characteristics eTable 6. Answers on the PSS-NICU eTable 7. Associations Between Parent Participation and Outcomes eReferences. [file jamanetwopen-e224514-s001.pdf]

Supplemental Online Content

van Veenendaal NR, van Kempen AAMW, Broekman BFP, et al. Association of a zero-separation neonatal care model with stress in mothers of preterm infants. *JAMA Netw Open.* 2022;5(3):e224514. doi:10.1001/jamanetworkopen.2022.4514

eMethods.

eFigure 1. Images of Care Single Family Rooms

eFigure 2. Image of Open Bay Unit With Standard Neonatal Care

eTable 1. Scale Properties and CO-PARTNER Tool

eTable 2. Response Rates of Mothers

eTable 3. Baseline Characteristics of Mothers With or Without Filled Out Questionnaires

eTable 4. Baseline Characteristics of Mothers With or Without Filled Out Questionnaires at Discharge

eTable 5. Missing Data in Baseline Characteristics

eTable 6. Answers on the PSS-NICU

eTable 7. Associations Between Parent Participation and Outcomes

eReferences.

This supplemental material has been provided by the authors to give readers additional information about their work.

© 2022 van Veenendaal NR et al. *JAMA Network Open.*

eMethods

The AMICA study

The AMICA study is a multicentre prospective observational cohort study on the association between an innovative FICare model in infants and their parents in a NICU level 2 context in the Netherlands. In this study a group of parents and infants who experienced family integrated care (FICare) in single family room units with complete couplet care for the mother-infant dyad, and a group who experienced standard care in open bay units are compared (the AMICA study[1], see eFigure 1a-d). In the AMICA study, preterm infants admitted for at least 7 days to one of the participating wards and their parents were included. The primary outcome in the AMICA study is the association between the FICare model and neurodevelopment of preterm infants. In the AMICA study, outcomes in parents (mothers and fathers separately) were also included as secondary outcomes in the short and longer term. We excluded families if mothers or fathers had severe psychosocial problems (for instance acute psychiatric illness or if a family was under supervision ofsocial services etc.), if death of a sibling occurred or if a congenital or metabolic syndrome was present in the infant.

Neonatal population in the Netherlands

Altered after van Veenendaal et al.[2].

In the Netherlands 17% of infants is born by cesarean section (8,1% primary and 7.9% secondary cesarean section) and approximately 70% of births occur in hospital (vs 30% in the community). In the Netherlands, 6.9% of infants are born preterm, and in the hospital region of Amsterdam the perinatal mortality rate (from 22 weeks of gestation up to 28 days after birth) is 0.56% (0.69% in the Netherlands) [3].

Different populations of preterm infants are defined within neonatal care in the Netherlands; Intensive care patients (IC), post-intensive high care patients, high-care (HC) patients and medium-care (MC) patients. *Intensive care infants*: Infants who need intensive care (e.g. cardiorespiratory support) are referred to a level 3 neonatal intensive care unit (NICU). In 2010, the Dutch guideline on perinatal practice in extremely premature delivery lowered the limit offering intensive care from 25+0 to 24+0 weeks of gestational age [4]. *Post-Intensive High Care infants*: Infants who are expected to need intensive care (gestational age <32 weeks and/or expected birth weight <1200 gram) are born in one of the ten level 3 NICUs. Once these preterm infants are stable and their actual weight is (around) 1000 gram or more, they are transferred to a hospital with a Post-Intensive-Care unit (level-2 Neonatal Ward). These Post-Intensive High Care infants often are still on non-invasive respiratory support, and/or have central venous catheters for parenteral nutrition or receive multiple medications. *High care infants*: Infants who are usually born in a level 2 neonatal ward, with a gestational age of at least 32 0/7 weeks and an expected birth weight >1200 grams. They also can be treated with non-invasive respiratory support, parenteral nutrition, central venous catheters and other medication. *Medium care infants*: Infants who are usually stable, growing infants without the need for cardiorespiratory monitoring or respiratory support, but can be treated with parenteral nutrition or medication by peripheral venous access. This group also consists of (near-) term small- or large for gestational age infants with glucose monitoring, stable infants treated with antibiotics

© 2022 van Veenendaal NR et al. *JAMA Network Open.*

for suspected perinatal infection, intravenous treatment of hypoglycemia or phototherapy for hyperbilirubinemia.

Sample size

The primary outcome of the AMICA study is neurodevelopment in preterm infants at 2 years of age.[1] We pre-stratified the study population towards infants born <32 weeks of gestation with a previous admission to a level 3 NICU and infants that were born >32 weeks of gestation. Within each gestational age group, we did a power calculation for the primary outcome of neurodevelopment. We calculated to have 64 experimental subjects and 128 control subjects with power 0.90 (1-β) at a significance level of 0.05 (α) with a true difference in the outcome of neurodevelopment of 2 years of ½ SD. To allow for 30% withdrawal we aimed to include 91 patients in group A and 182 patients in group B per risk group (post-intensive care versus inborn infants). A total of 546 infants who were hospitalised and their parents were expected to be included in this study.

For this study we calculated the power for the outcome on stress in mothers *post-hoc*.[6] The group sample sizes of 124 and 115 achieved 93% power to detect a difference of -9.8 between the null hypothesis that both group means are 47.2 and the alternative hypothesis that the mean of group 2 is 57.0 with known group standard deviations of 22.2 and 22.2 and with a significance level (alpha) of 0.05 using a two-sided two-sample t-test.

Characteristics of scales used in this study

Stress

The Parental Stressor Scale:Neonatal Intensive Care Unit (PSS-NICU) is a scale to measure parental perceptions of stressors associated with the hospitalisation of their child. It measures parents' perceptions of stressors arising from the physical and emotional environment. It takes in account the infant's behaviour and appearance, parental role alterations, and the sights and sounds of the environment[7,8].

The PSS-NICU questionnaire has 3 subdomains and measures the degree of stress experienced by parents during hospitalization related to alterations in their parental role, the appearance and behavior of their infant, and sights and sounds of the unit. Parents rate their experiences on a 5-point rating scale ranging from "not at all stressful" (0) to "extremely stressful" (5). In an update of the tool in 2007, sights and sounds of the environment (5 items) were combined with infant's appearance subscale (14 items) and scored as one subscale and Parental Role Alteration as the second subscale (7 items).[9] If mothers did not experience the stressor, we transformed the score to "0".[10] The PSS:NICU has been translated into Dutch and Cronbach alpha of this questionnaire is been shown to be 0.89-0.94[8].

Depression and anxiety

Depression and anxiety scores at discharge were measured with the Hospital Anxiety and Depression scale, which has previously been validated in the Dutch population. It contains two 7-item scales:

© 2022 van Veenendaal NR et al. *JAMA Network Open.*

one for anxiety and one for depression both with a score range of 0-21. It has been validated in the Dutch population before in ages 16 to 65 years[11].

Parent- and infant bonding
The Postpartum Bonding Questionnaire (PBQ), was devised by Brockington et al. (2001) as a screening instrument to detect bonding problems in obstetric and primary care services [12,13]. The PBQ is a 25- item scale reflecting a mother's feelings or attitudes towards her baby (e.g. ''I feel close to my baby'', ''My baby irritates me''). Participants rate how often they agree with these statements on a 6-point Likert scale ranging from always (score=0) to never (score=5) with low scores denoting good bonding. The PBQ has four subscales which reflect impaired bonding (Scale 1) (12 items, ranging from 0 to 60), rejection and anger (Scale 2) (7 items, scores ranging from 0 to 35), anxiety about care (Scale 3) (4 items, scores ranging from 0 to 20) and risk of abuse (Scale 4) (2 items, scores ranging from 0 to 10). Scale 1 (impaired bonding) has a sensitivity of 0.93 and a specificity of 0.85 in detecting mothers with a bonding disorder.

Parental empowerment and satisfaction
Parent satisfaction was measured using the EMpowerment of PArents in THe Intensive Care - Neonatology questionnaire[14]. This questionnaire was developed and tested in a single center in the Netherlands, and available in Dutch. The domains covered are: Information (14 statements); Care and Treatment (20 statements); Parental Participation (nine statements); Organization (11 statements); and Professional Attitude (13 statements). The 57 statements divided in five domains provide a conceptualization of parent satisfaction within the neonatal ward from a family-centred care perspective[14].

Parental Self Efficacy
The Perceived (Maternal) Parenting Self-Efficacy (PMP-SE) tool, was used to measure perceived parental self-confidence when caring for the infant admitted to the Neonatal Ward [15]. The internal consistency reliability of the Perceived Maternal Parenting Self-Efficacy tool is 0.91, external/test-retest reliability is 0.96. A total of four conceptually unique subscales of parenting are: "Care taking procedures" (parents' perceptions of their ability to perform the activities and tasks related to the baby's basic needs like feeding). "Evoking behaviour(s)" (perceptions in their ability to elicit a change in the baby's behaviour, for example, soothing the baby). "Reading behaviour(s) or signalling" (perceptions in their ability to understand and identify changes in their baby's behaviour, for example, 'I can tell when my baby is sick'). "Situational beliefs"(parents' beliefs about their ability to judge their overall interaction with the baby). Responses to each item were recorded on a four point Likert scale ranging from 'strongly disagree' (score 1) to 'strongly agree' (score 4). A low score on this scale indicates a low parental self-efficacy.

CO-PARTNER tool
We previously validated a measurement tool on parent participation in neonatal care (the CO-PARTNER tool), which includes 6 domains: 1) daily care 2) medical care 3) information gathering 4) advocacy and leadership 5) time spent with infant 6) closeness and comforting the infant.[16] Parents fill out the scale reflecting on a set time-frame by the researchers. In this study, parents reflected on their participation during infant hospital stay up to discharge. Total scores per domain were obtained by summing scores. For Domain 1, 2 and 6 we calculated 0 for 'the nurse does this', 1 for

© 2022 van Veenendaal NR et al. *JAMA Network Open.*

‘the nurse and I do this together’ and 2 for ‘I do this independently’ (minimum scores 0 to 22, 8 and 14 respectively). For domain 3 and 4 ‘yes’ was scored as 1, and ‘no’ as 0 (minimum scores 0 to 3). Non-applicable items were transformed to 0 (no participation in this item). For the domain Time Spent with Infant (3 items) quartiles were calculated resulting in 0 to 4 score (total score in domain 5 minimum 0 maximum 12). A total participation score was obtained by summing all domain scores. Minimum total scores were 0 and maximum 62.

Confounders and effect modifiers

Potential confounders and effect modifiers were identified from the literature and assessed using statistical analyses. We considered socio-economic class (including education and employment status), family composition (single parent vs co-parenting), older/other infants in the family, stress at birth, gestational age of infant, singleton status and mode of delivery. If the beta-regression coefficient differed at least 10% in regression analyses, this was used as an indication of statistical confounding, and the variable was included in the adjusted model. If collinearity was present, the strongest confounder (largest change in crude beta-coefficient) was used to adjust for.

© 2022 van Veenendaal NR et al. *JAMA Network Open.*

eFigure 1. Images of Care Single Family Rooms

A. Single family room with complete couplet care for the mother-infant dyad

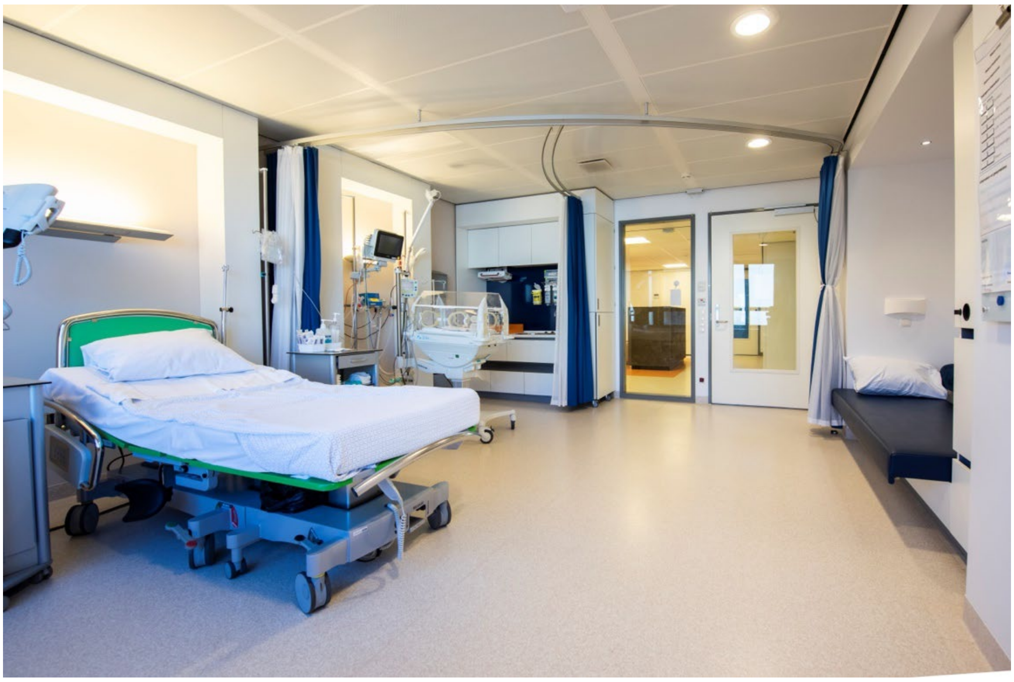

A Single-Family Room for highly complex maternity and neonatal Level 2 Care. Women and their newborns will remain in this suite for as long as both require specialized care, or at least for 7 days if the newborn requires specialized care. Fathers, too, can be present continuously. If after 7 days and one of them no longer needs specialized care, the woman and the newborn are transferred to a smaller single-family room, a room for highly complex maternity care and neonatal level 1 care or a room for neonatal level 2 care. All single-family rooms provide rooming-in facilities for one parent/partner.[5] Copyrights Audiovisuele Zaken, OLVG, Amsterdam, The Netherlands, June 2020.

© 2022 van Veenendaal NR et al. *JAMA Network Open.*

B. Single family room for neonatal level 2 care

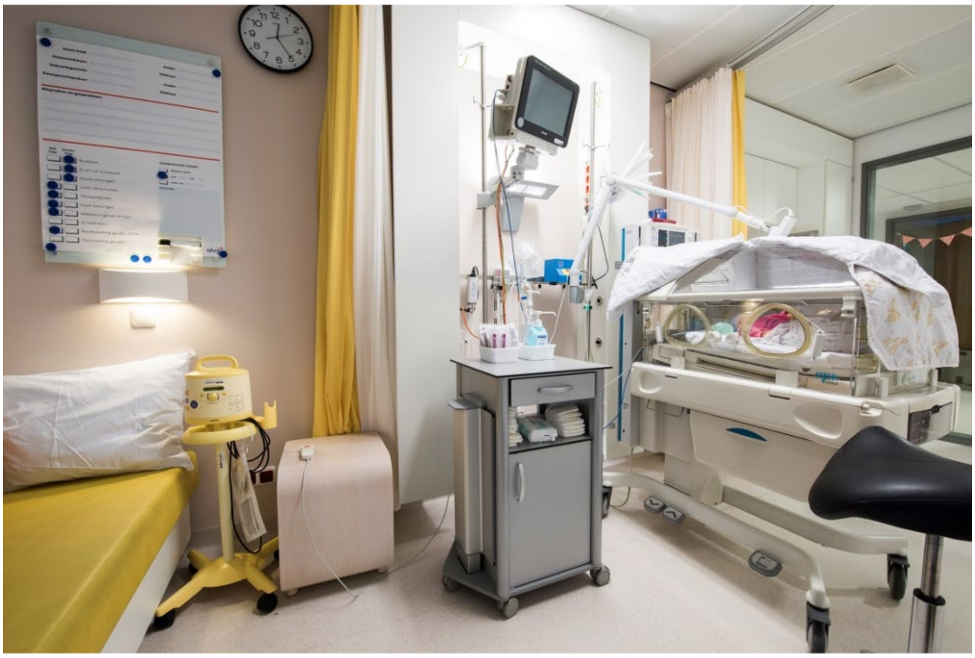

Copyrights Audiovisuele Zaken, OLVG, Amsterdam, The Netherlands, June 2020.

C. Family participation in the single family room

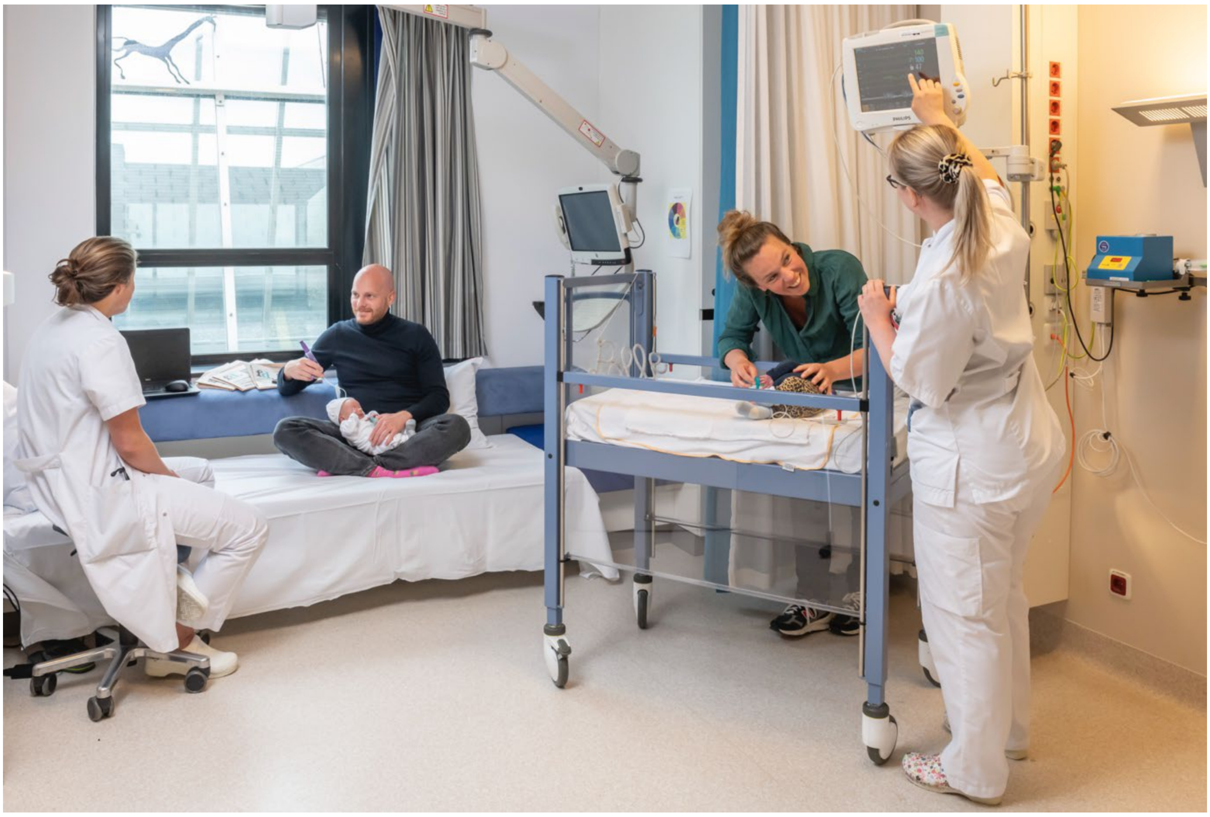

Depicted is a family with twin infants born at a gestational age of 32 weeks, together with a doctor and nurse specialized in neonatal care. The family stays continuously together in a single family room in our integrated neonatal-maternity ward. This enables both parents to participate, as equal partners in the medical team, in the care and medical decision making for their infants during hospital stay.
Copyrights Audiovisuele Zaken, OLVG, Amsterdam, The Netherlands, June 2020.

eFigure 2. Image of Open Bay Unit With Standard Neonatal Care

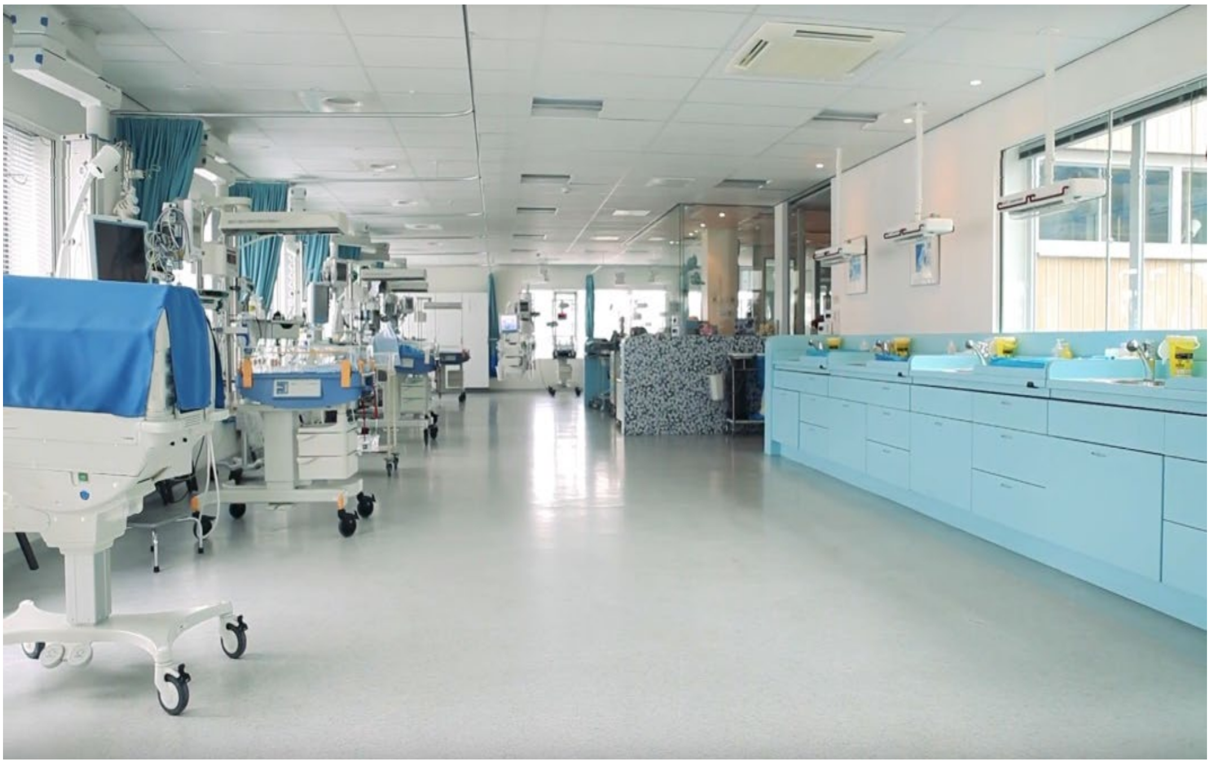

Copyrights Audiovisuele Zaken, OLVG, Amsterdam, The Netherlands, June 2020.

eTable 1. Scale Properties and CO-PARTNER Tool

A. Scale properties

	Questionnaire	Minimum score	Maximum score	Internal consistency/ Cronbach's alpha (reference)
Stress	PSS-NICU	0	130	0·89-0·94 [8,9]
	Sights and sounds, behaviour of the infant	0	95	0.92 [9]
	Parental role alteration	0	35	0.92 [9]
Anxiety and depression	HADS	0	42	0.71-0.90 [11]
Self-efficacy	PMP-SE	20	80	0.91 [15]
Mother infant bonding	PBQ	0	125	0.87-0.78 [17]
Satisfaction with care (median scores over all items)	EMPATHIC-N	1	6	082 -0.95 [14]
Collaboration and participation in neonatal care	CO-PARTNER	0	62	NA [16]
	**Domain 1** *Participation in daily care*	0	22	0.934 [16]
	**Domain 2** *Participation in medical care*	0	8	0.558 [16]
	**Domain 3** *Information gathering*	0	3	0.745 [16]
	**Domain 4** *Advocacy and leadership*	0	3	0.855 [16]
	**Domain 5** *Time spent with infant*	0	12	0.839 [16]
	**Domain 6** *Comforting the child*	0	14	0.871 [16]

EMATHICN-N: EMpowerment of PArents in THe Intensive Care- Neonatology, HADS: hospital anxiety and depression score, PBQ: postpartum bonding questionnaire, PMP-SE: *The Perceived (Maternal) Parenting* Self-Efficacy, PSS-NICU: parental stress scale – neonatal intensive care unit

© 2022 van Veenendaal NR et al. *JAMA Network Open.*

B. CO-PARTNER tool (after van Veenendaal et al.[16])

Activity	Response
**Domain 1. Daily Care**	
1.Bath my child/clean my child with a washcloth.	○ The nurse does this<br>○ I do this together with the nurse<br>○ I do this independently (without the help of the nurse)<br>○ This is not applicable
2. Change my child's diaper.	○ The nurse does this<br>○ I do this together with the nurse<br>○ I do this independently (without the help of the nurse)<br>○ This is not applicable
3. Feed my child (breast or bottle).	○ The nurse does this<br>○ I do this together with the nurse<br>○ I do this independently (without the help of the nurse)<br>○ This is not applicable
4. Change my child's clothing.	○ The nurse does this<br>○ I do this together with the nurse<br>○ I do this independently (without the help of the nurse)<br>○ This is not applicable
5. Get my child out of the incubator/cradle.	○ The nurse does this<br>○ I do this together with the nurse<br>○ I do this independently (without the help of the nurse)<br>○ This is not applicable
6. Give my child medication.	○ The nurse does this<br>○ I do this together with the nurse<br>○ I do this independently (without the help of the nurse)<br>○ This is not applicable
7. Weigh my child.	○ The nurse does this<br>○ I do this together with the nurse<br>○ I do this independently (without the help of the nurse)<br>○ This is not applicable

© 2022 van Veenendaal NR et al. *JAMA Network Open.*

8. Keep track of output (urination and defecation) of my child	○ The nurse does this<br>○ I do this together with the nurse<br>○ I do this independently (without the help of the nurse)<br>○ This is not applicable
9. Measure the temperature of my child.	○ The nurse does this<br>○ I do this together with the nurse<br>○ I do this independently (without the help of the nurse)<br>○ This is not applicable
10. Keep track of my child's weight.	○ The nurse does this<br>○ I do this together with the nurse<br>○ I do this independently (without the help of the nurse)<br>○ This is not applicable
11. Keep track of drinking and my child's feeds.	○ The nurse does this<br>○ I do this together with the nurse<br>○ I do this independently (without the help of the nurse)<br>○ This is not applicable
**Domain 2. Medical Care**	
12. Give tube feeding to my child.	○ The nurse does this<br>○ I do this together with the nurse<br>○ I do this independently (without the help of the nurse)<br>○ This is not applicable
13. Look at my child's monitor and handling accordingly (e.g. stimulating during a bradycardia).	○ The nurse does this<br>○ I do this together with the nurse<br>○ I do this independently (without the help of the nurse)<br>○ This is not applicable
14. Regulate the visiting of others to my child.	○ The nurse does this<br>○ I do this together with the nurse<br>○ I do this independently (without the help of the nurse)<br>○ This is not applicable
15. Participate in the daily rounds with the doctor.	○ The nurse does this<br>○ I do this together with the nurse<br>○ I do this independently (without the help of the nurse)<br>○ This is not applicable

© 2022 van Veenendaal NR et al. *JAMA Network Open.*

Domain 3. Acquiring Information	
16. Did you ask healthcare professionals information on the health of your child?	○ Yes<br>○ No
17. Did you ask the healthcare professionals for information about your child for times when you were not present?	○ Yes<br>○ No
18. Did you talk with another parent about your experiences?	○ Yes<br>○ No
**Domain 4. Parent Advocacy**	
19. I stood up for my child; I told somebody to do something in the care of my child.	○ Yes<br>○ No
20. I stood up for my child; I told somebody NOT to do something in the care of my child; I gave boundaries	○ Yes<br>○ No
21. I gave an explanation on the daily routines of my child to a healthcare professional.	○ Yes<br>○ No
**Domain 5. Time Spent with Infant**	
22. On average, how many hours were you present in the hospital with your child?	Number of hours per day:
23. On average, how many hours a day do you have contact with your child?	Number of hours per day:
24. On average, how many hours were you really close with your child?	Number of hours per day:
**Domain 6. Closeness and Comforting the Infant**	
25. Hold/rock/cuddle my child.	○ The nurse does this<br>○ I do this together with the nurse<br>○ I do this independently (without the help of the nurse)<br>○ This is not applicable

© 2022 van Veenendaal NR et al. *JAMA Network Open.*

26. Comfort my child whenever he/she needs it.	○ The nurse does this<br>○ I do this together with the nurse<br>○ I do this independently (without the help of the nurse)<br>○ This is not applicable
27. Kangaroo care / skin to skin contact.	○ The nurse does this<br>○ I do this together with the nurse<br>○ I do this independently (without the help of the nurse)<br>○ This is not applicable
28. Be together with my child, be close with my child. (intimate time).	○ The nurse does this<br>○ I do this together with the nurse<br>○ I do this independently (without the help of the nurse)<br>○ This is not applicable
29. Be together with my child (be present).	○ The nurse does this<br>○ I do this together with the nurse<br>○ I do this independently (without the help of the nurse)<br>○ This is not applicable
30. Soothe my child during a painful procedure (for instance drawing blood).	○ The nurse does this<br>○ I do this together with the nurse<br>○ I do this independently (without the help of the nurse)<br>○ This is not applicable
31. Recognize my child's signals.	○ The nurse does this<br>○ I do this together with the nurse<br>○ I do this independently (without the help of the nurse)<br>○ This is not applicable

© 2022 van Veenendaal NR et al. *JAMA Network Open.*

eTable 2. Response Rates of Mothers

	**Non-missing (Filled out questionnaires) *<br>N= 239/296 (80.7%)**		p-value
	FICare	Standard Care	
Mothers <32 (No. (%))	60 / 68 (88.2%)	25 / 35 (71.4%)	
Mothers >32 (No. (%))	64 / 73 (87.7%)	90 / 120 (75.0%)	
Mothers total (No. (%))	124 / 141 (87.9%)	115 / 155 (74.2%)	<0.004

*** at discharge and/or admission and/or 3 months of age, n: number, NA: not applicable,**

© 2022 van Veenendaal NR et al. *JAMA Network Open.*

eTable 3. Baseline characteristics of mothers with or without filled out questionnaires

	**Filled out questionnaire (n=239)**	**Did not fill out questionnaire (N=57)**	
FICare (No. (%))	124 (56.1)	17 (29.8)	0.004
Gestational age (weeks, median (IQR))	33+2 (30+4 – 34+6)	33+2 (31+0 – 35+0)	0.869
Inborn (No. (%))	133 (55.6)	30 (52.6)	0.792
Singleton status (No. (%))	34 (14.2)	13 (22.8)	0.164
Paid work (No. (%))	176	NA	NA
Identifies with Dutch background (No. (%))	176	NA	NA
Total length of stay in hospital (birth to discharge, days, median (IQR))	23 (15 – 46)	25 (13 – 43)	0.382
Length of stay in level 2 (admission to discharge, median (IQR))	23 (15 – 37)	23 (13 – 34)	0.353

FICare: family integrated care, IQR: interquartile range, N: number, NA: not applicable,

© 2022 van Veenendaal NR et al. *JAMA Network Open.*

eTable 4. Baseline characteristics of mothers with or without filled out questionnaires at discharge

	**Filled out questionnaires at discharge (n=198)**	**Did not fill out questionnaire at discharge (n=41)**	
FICare (No. (%))	105 (53.0)	19 (46.3)	0.543
Gestational age (weeks, median (IQR))	33+3 (31+0 – 34+6)	31+3 (28+2 – 34+3)	0.013
Inborn (No. (%))	113 (57.1)	20 (48.8)	0.424
Singleton status (No. (%))	26 (13.1)	7 (17.1)	0.413
Pre-eclampsia (No. (%))	41 (20.7)	9 (22.0)	0.943
Maternal HELLP syndrome (No. (%))	11 (5.6)	1 (2.4)	0.696*
Vaginal delivery (No. (%))	103 (52.0)	21 (51.2)	0.331
Paid work (No. (%))	162 (81.8)	14 (34.1)	0.005
Work hours per week (mean (SD))	36 (32 – 40)	34.5 (24.6 – 40)	0.220
Identifies with Dutch background (No. (%))	161 (81.3)	15 (36.6)	0.0009
Total length of stay in hospital (birth to discharge, days, median (IQR))	22 (15 – 43)	38 (20 – 83)	0.0077
Length of stay in level 2 (admission to discharge, median (IQR))	22 (14 – 35)	31 (19 – 50)	0.0142
Length of stay in level 3 (admission to discharge, median (IQR))	0 (0 – 6)	2 (0- 21)	0.061
GA <32 weeks (No. (%))	63 (31.8)	22 (53.7)	0.013
University degree (No. (%))	179 (90.4)	18 (43.9)	0.001
HADS at admission (median (IQR))	10 (7 – 14)	13 (10 – 16)	0.066
PBQ at admission (median (IQR))	8 (4.5 – 13.5)	5.5 (2 – 8.8)	0.070
PMP at admission (mean (SD))	60.2 (9.9)	62.8 (11.3)	0.393
PSS at admission (mean (SD))	54.8 (21.6)	59.6 (24.9)	0.466
Smoking during pregnancy (No. (%))	4 (2.0)	1 (2.4)	0.450*
Use of drugs (No. (%))	16 (8.1)	3 (7.3)	0.439*
Use of psychotropic drugs (No. (%))	4 (2.0)	1 (2.4)	1.000*
Alcohol use during pregnancy (No. (%))	1 (0.5)	0	1.000

*Fisher exact test, n: number

Variables associated with missing data at discharge were infant gestational age, work status of the mother, cultural background, total length of stay in the hospital, and education level, and used for the multiple imputation model.

© 2022 van Veenendaal NR et al. *JAMA Network Open.*

eTable 5. Missing data in baseline characteristics

Characteristic	FICare group (n=124), missing (No (%))	SNC group (n=115), missing (No (%))
Age	0	1 (0.9)
University degree	11 (8.9)	15 (13.0)
Paid job	11 (8.9)	15 (13.0)
Identifies with Dutch background	9 (7.3)	13 (11.3)
Stress of pregnancy	9 (7.3)	17 (14.8)
Stress of birth	11 (8.9)	19 (16.5)
Pre-eclampsia	4 (2.4)	1 (0.9)
HELLP syndrome	0	3 (2.6)
Use of psychofarmaca	0	0
Gestational age	0	0
Born < 32 weeks of gestation	0	0
Inborn infant (born in level 2 hospital)	0	0
Singleton	0	0
Vaginal delivery	0	0
First child upbringing	11 (8.9)	20 (17.4)
Plan for upbringing *Together with partner*	11 (8.9)	21 (18.3)
Depression and anxiety score at admission	70 (56.5)	88 (76.5)
Total stress at admission	69 (55.6)	86 (74.8)

© 2022 van Veenendaal NR et al. *JAMA Network Open.*

eTable 6. Answers on the PSS-NICU

**Question**	**NA / 0 (n)**	**1 (n)** Not at all stressful	**2 (n)** A little stressful	**3 (n)** Moderately stressful	**4 (n)** Very stressful	**5 (n)** Extremely stressful	**Total answers (n)**
1 The presence of monitors and equipment	15	49	67	40	19	1	191
2 The constant noises of monitors and equipment	41	32	57	39	16	6	191
3 The sudden noises of monitor alarms	37	16	41	48	37	12	191
4 The other sick babies in the room	87	46	23	21	11	3	191
5 The large number of people working in the unit	14	102	41	18	13	2	190
6 Tubes and equipment on or near my baby	28	47	57	35	18	3	188
7 Bruises, cuts or incisions on my baby	71	16	33	28	28	10	186
8 The unusual color of my baby (for example looking pale or yellow jaundiced)	59	29	44	26	17	10	185
9 My baby's unusual or abnormal breathing patterns	46	10	44	29	41	14	184
10 The small size of my baby	18	53	54	31	22	5	183
11 The wrinkled appearance of my baby	88	64	15	9	7	1	184
12 Having a machine (respirator) breathe for my baby	99	10	20	26	22	6	183
13 Seeing needles and tubes put in my baby	41	12	45	31	35	19	183
14 My baby being fed by an intravenous line or tube	21	74	48	29	8	3	183
15 When my baby seemed to be in pain	19	6	32	44	52	29	182
16 When my baby looked sad	14	16	45	39	47	21	182
17 The limp and weak appearance of my baby	80	12	24	23	28	15	182
18 Jerky or restless movements of my baby	21	28	59	47	21	6	182
19 My baby not being able to cry like other babies	121	28	19	8	3	2	181
20 Being separated from my baby	46	15	16	34	43	34	188
21 Not feeding my baby myself	52	37	28	32	25	13	187
22 Not being able to care for my baby myself (for	81	29	25	23	18	12	188

© 2022 van Veenendaal NR et al. *JAMA Network Open.*

example, diapering, bathing)							
23 Not being able to hold my baby when I want	58	15	29	28	34	24	188
24 Feeling helpless and unable to protect my baby from pain and painful procedures	31	19	38	32	38	30	188
25 Feeling helpless about how to help my baby during this time	29	23	42	40	31	22	187
26 Not having time alone with my baby	61	30	37	32	18	9	187

n: number

© 2022 van Veenendaal NR et al. *JAMA Network Open.*

eTable 7. Associations between parent participation and outcomes

Outcome	Beta (95%CI)	p-value	Adjusted beta (95%CI)[a]	p-value
Depression/anxiety	-0.024 (-0.039; -0.009)[b]	0.0018	-0.024 (-0.038; -0.009)[b]	0.002
Impaired mother-infant bonding	-0.031 (-0.049; -0.014)[b]	0.0006	-0.030 (-0.047; -0.013)[b]	0.0007
Self-efficacy	0.343 (0.161; 0.526)	0.0003	0.330 (0.150; 0.510)	0.0004
Satisfaction of care	0.004 (-0.005; 0.013)	0.357	0.007 (-0.002;0.015)	0.145

All outcomes are pooled estimates from multiple imputed datasets, [a]adjusted for: Gestational age, gemelli status, education, age, Dutch background, singleton status, stress at birth and first child upbringing. NA: not applicable [b]after ln transformation

© 2022 van Veenendaal NR et al. *JAMA Network Open.*

eReferences.

1. van Veenendaal NR, van Kempen AAMW, Maingay F, Recourt-Vollebregt M, van der Schoor SRD, van Goudoever J. Family Integrated Care in the Neonatal Ward - the AMICA study. https://www.trialregister.nl/trial/6175. Published 2017. Accessed March 19, 2020.
2. van Veenendaal NR, van der Schoor SRD, Heideman WH, et al. Family integrated care in single family rooms for preterm infants and late-onset sepsis: a retrospective study and mediation analysis. *Pediatr Res*. 2020. doi:10.1038/s41390-020-0875-9
3. Perined, van Dijk A, Dijs-Elsinga J, et al. *Perined. Perinatale Zorg in Nederland 2016*. Utrecht; 2016. https://assets.perined.nl/docs/7935f9c6-eaac-4f59-a150-307ae04efa27.pdf. Accessed April 17, 2018.
4. de Laat MWM, Wiegerinck MM, Walther FJ, et al. Richtlijn "Perinataal beleid bij extreme vroeggeboorte". *Ned Tijdschr Geneeskd*. 2010;154.
5. Stelwagen MA, van Kempen AAMW, Westmaas A, Blees YJ, Scheele F. Integration of Maternity and Neonatal Care to Empower Parents. *J Obstet Gynecol Neonatal Nurs*. 2020;49(1):65-77. doi:10.1016/j.jogn.2019.11.003
6. Machin D, Campbell MJ, Tan SB, Tan SH. *Sample Size Tables for Clinical Studies: Third Edition*. Wiley-Blackwell; 2009. doi:10.1002/9781444300710
7. Miles MS, Brunssen SH. Psychometric properties of the parental stressor scale: Infant hospitalization. *Adv Neonatal Care*. 2003. doi:10.1016/S1536-0903(03)00138-3
8. Miles MS, Funk S, Carlson J. Parental Stressor Scale: Neonatal Intensive Care Unit. *Nurs Res*. 1993;42(3):148-152.
9. Miles MS, Holditch-Davis D, Schwartz TA, Scher M. Depressive symptoms in mothers of prematurely born infants. *J Dev Behav Pediatr*. 2007. doi:10.1097/01.DBP.0000257517.52459.7a
10. Schappin R, Wijnroks L, Uniken Venema MMAT, Jongmans MJ. Rethinking Stress in Parents of Preterm Infants: A Meta-Analysis. *PLoS One*. 2013;8(2). doi:10.1371/journal.pone.0054992
11. Spinhoven P, Ormel J, Sloekers PPA, Kempen GIJM, Speckens AEM, Van Hemert AM. A validation study of the Hospital Anxiety and Depression Scale (HADS) in different groups of Dutch subjects. *Psychol Med*. 1997. doi:10.1016/S0163-8343(03)00043-4
12. Brockington IF, Fraser C, Wilson D. The Postpartum Bonding Questionnaire: A validation. *Arch Womens Ment Health*. 2006;9(5):233-242. doi:10.1007/s00737-006-0132-1
13. Brockington IF, Oates J, George S, et al. A screening questionnaire for mother-infant bonding disorders. *Arch Womens Ment Health*. 2001. doi:10.1007/s007370170010
14. Latour JM, Duivenvoorden HJ, Hazelzet JA, Van Goudoever JB. Development and validation of a neonatal intensive care parent satisfaction instrument. *Pediatr Crit Care Med*. 2012;13(5):554-559. doi:10.1097/PCC.0b013e318238b80a
15. Barnes CR, Adamson-Macedo EN. Perceived Maternal Parenting Self-Efficacy (PMP S-E) tool: Development and validation with mothers of hospitalized preterm neonates. *J Adv Nurs*. 2007;60(5):550-560. doi:10.1111/j.1365-2648.2007.04445.x
16. van Veenendaal NR, Auxier JN, van der Schoor SRD, et al. Development and psychometric evaluation of the CO-PARTNER tool for collaboration and parent participation in neonatal care. Alves E, ed. *PLoS One*. 2021;16(6):e0252074. doi:10.1371/journal.pone.0252074
17. Van Bussel JCH, Spitz B, Demyttenaere K. Three self-report questionnaires of the early mother-to-infant bond: Reliability and validity of the Dutch version of the MPAS, PBQ and MIBS. *Arch Womens Ment Health*. 2010;13(5):373-384. doi:10.1007/s00737-009-0140-z

© 2022 van Veenendaal NR et al. *JAMA Network Open.*
